# Biliary tract large cell neuroendocrine carcinoma: current evidence

**DOI:** 10.1186/s13023-019-1230-2

**Published:** 2019-11-21

**Authors:** Riva Raiker, Aman Chauhan, Hassan Hasanein, Grant Burkeen, Millicent Horn, Janeesh Veedu, Cory Vela, Susanne Arnold, Jill Kolesar, Lowell Anthony, B. Mark Evers, Michael Cavnar

**Affiliations:** 1 Department of Internal Medicine, University of Kentucky, Lexington, KY USA; 2 Department of Pharmacology, University of Kentucky, Lexington, KY USA; 3 Department of Pharmacy Practice and Science, College of Pharmacy, University of Kentucky, Lexington, KY USA; 4 Department of Surgery, University of Kentucky, Lexington, KY USA; 5 Markey Cancer Center, University of Kentucky, Lexington, KY USA

**Keywords:** Neuroendocrine, Large cell neuroendocrine carcinomas, Biliary tract

## Abstract

**Background:**

Primary neuroendocrine carcinomas of the gallbladder and biliary tract are rare, with pure large cell neuroendocrine carcinomas (LCNEC) being exceedingly rare and with a particularly poor prognosis.

**Methods:**

We performed a review of published data on biliary tract large cell neuroendocrine carcinomas in PubMed.

**Results:**

Preliminary search revealed over 2000 results but we found only 12 cases of pure large cell neuroendocrine carcinomas of biliary tract noted in literature to date. Because it commonly presents with non-specific symptoms of abdominal pain and jaundice, diagnosis is made after resection with histo-pathological and immunohistochemical analysis. These cancers are particularly aggressive with high recurrence rates, most often presenting with metastasis to regional lymph nodes and/or the liver resulting in a poor prognosis. Overall, complete surgical excision with systemic chemotherapy is the treatment mainstay. If the cancer is unresectable due to multiple metastases, medical management with systemic chemotherapy is the primary treatment modality.

**Conclusion:**

The prognosis of hepatobiliary LCNEC remains poor with median survival of only 11 months from initial diagnosis. Studies focusing on high grade neuroendocrine carcinoma are needed to enhance our understanding of biology and therapeutics in this rare but aggressive cancer.

## Background

Neuroendocrine neoplasms (NEN) are a heterogeneous group of rare tumors that account for approximately 2% of all malignancies and 0.5% of all newly diagnosed malignancies [1]. Despite being a rare disease, the incidence is on the rise [2, 3]. Although they occur sporadically, NENs can occur in association with hereditary syndromes. NENs often originate from neuroendocrine cells, which are specialized cells that receive neuronal input and synthesize and secrete hormones in response to that stimulus, thereby connecting the nervous and endocrine systems of the body. Due to this secretory function, neuroendocrine tumors (NETs) can cause a variety of clinical syndromes depending upon the hormone released. Neuroendocrine cells are located throughout the body, most notably in the hypothalamus and pituitary gland, as well as the gastrointestinal and respiratory tract; thus, NETs can originate anywhere in the body.

The classification, grading, and staging of NEN can vary based on the primary tumor site, proliferative rate and the extent of invasion and spread. The most current classification, based on the European Neuroendocrine Tumor Society and the World Health Organization (WHO), broadly separates NENs into well-differentiated and poorly-differentiated neoplasms. Well-differentiated neoplasms are called neuroendocrine tumors (NETs) and can be either low grade (G1), with a mitotic count < 2 per 10 high power field (HPF) and < 3% Ki-67 index, or intermediate grade (G2), with mitotic activity of 2–20 per 10 HPF and 3–20% Ki-67 index. Recently WHO added a new category of well-differentiated grade 3 NETs to pancreatic NETs. Poorly differentiated NENs, also called neuroendocrine carcinomas (NEC), are profoundly aggressive and considered high grade (G3), with > 20% Ki-67 index. Non-well-differentiated high grade neuroendocrine carcinoma can be further divided into either small cell, large cell, or mixed NEC. Neoplasms containing > 30% of both neuroendocrine and non-neuroendocrine components are called mixed neuroendocrine-non-neuroendocrine neoplasms (MiNEN) [4].

Large cell neuroendocrine carcinomas are part of the high-grade subset of NEC and are exceedingly rare and aggressive. Histologically these tumors have similarities to well-differentiated NETs with a trabecular or organoid growth pattern, rosette formations, “salt and pepper” chromatin, and/or peripheral palisading, as well as features of poorly-differentiated NECs with high mitotic activity and large areas of necrosis [5–7]. Unlike small cell neuroendocrine carcinomas (SCNEC), cells in LCNEC are generally two to three times larger, have prominent nucleoli, and a lower nuclear-to-cytoplasmic ratio [5, 7, 8]. Diagnosis is often confirmed with positive immunohistochemical neuroendocrine markers, most commonly synaptophysin, chromogranin A, or CD56 but can also include protein cell product 9.5, neuron-specific enolase (NSE), and Leu 7 [5, 7]. Thoracic LCNECs form the most common site of origin followed by the gastrointestinal tract. Little is known about the management of LCNECs arising from other primary locations.

Primary NETs of the hepatobiliary tract are rare and account for only 2% of all gallbladder and biliary tract tumors [9, 10]. Pure LCNECs of the biliary tract are exceedingly limited with only 12 cases identified in the literature by our review to date, as most cases reported have mixed features. It is unclear how NECs originate in the gallbladder or biliary tract as neuroendocrine cells are not ordinarily present in the mucosa, which is likely why these cancers are particularly rare. It has been hypothesized that these may arise from an undifferentiated stem cell or in the setting of chronic inflammation leading to metaplasia and subsequent malignant conversion [11–13]. These tumors are invasive and often present with lymph node or distant metastasis causing a poor overall prognosis that appears to be similar or worse than that of SCNEC [13–15]. There is very limited data regarding management of hepatobiliary LCNEC. This review will analyze all published materials to date on this particular subset of pure LCNECs to help understand this rare and aggressive form of cancer.

## Methods

We performed a systematic review of published data on biliary tract large cell neuroendocrine carcinoma. The search words used were: “management of large cell neuroendocrine carcinoma;” “large cell neuroendocrine carcinoma;” “hepatic large cell neuroendocrine carcinoma;” hepatobiliary large cell neuroendocrine carcinoma, large cell neuroendocrine carcinoma of gallbladder, large cell neuroendocrine carcinoma of common bile duct, and large cell neuroendocrine carcinoma of biliary tract. A total 2183 articles were found in PUBMED with only 21 articles relevant to our topic. Of the 21 articles, 12 were pertinent to management of hepatobiliary large cell neuroendocrine carcinoma with relevant clinical details of patients abstracted (Table 1). The articles were reviewed for study variables including tumor primary site, age at diagnosis, patient gender, presenting symptoms, presence of metastasis, treatment method (chemotherapy, surgery, radiation), and outcomes.

**Table 1:** Pure Large Cell Hepatobiliary Neuroendocrine Carcinoma

Case	Ref.	Age (yr)	Sex	Site	Presentation	Metastasis	Chemo	Surgery	Outcome^**a**^
1	[6]	65	M	GB	Symptomatic Cholelithiasis	Liver (4mn after CCY)	Chemo NS	CCY LR	Died 18 mo
2	[16]	55	M	GB	Abdominal Pain + Jaundice	Multiple LN	Chemo NS (1 cycle)	Celiac LN Bx; unresect	Died 1 mo
3	[16]	67	F	GB	Abdominal Pain	Liver	Chemo NS (3 cycles)	Liver/GB Bx, unresect	Died 10 mo
4	[15]	58	F	GB	18mo hx Suggestive of Gallstone	Regional LN	Cis + ETP (5 cycles, adjuvant)	CCY → 2 mo later radical GB Bed Clearance, Partial LR, CBD excision, and LND up to coeliac nodes	Alive (16 mo post-CCY)
5	[17]	64	F	GB	Abdominal Pain	Liver / Brain	Hepatic Arterial Chemo Infusion: Cis/ETP + CAV + Pre-op 3D RT Post-op Chemo with Cis/ETP	RHep Triseg; Cerebell +γ-knife	Died 69mo after 1st diagnosis (3 yr without recurrence since the last γ-knife)
6	[10]	67	F	GB CBD	Abdominal Pain + Jaundice	Multiple LN; Liver; Lung; Vertebra, Peritoneum	Cis + ETP (1 cycle)	Unresect	Died < 1 mo
7	[9]	56	M	GB	Exophthalmos	Liver; Ileum; Kidneys; Adrenal Glands; Diaphragm; Epicardium; Left Orbit; Abdominal LN	Chemo-radiotherapy NS	Unresect	Died 36 mo
8	[18]	64	M	GB	Abdominal Fullness	Liver; Multiple LN; Bone	Cis + TXT→ CBDCA + TXT	Axillary LN + GB Bx Unresect	Died 22 mo
9	[8]	76	M	CBD	Jaundice	Multiple LN; GB	None	RHepLob; Bile Duct Resection, CCY	Died 21 days
10	[13]	75	F	CBD	Nausea Jaundice	LN; → recurrence at HJ site; Liver; Portocaval Area	5-FU+ EPR + CIS (adjuvant)	CBD excision with LN dissection	Died 12 mo
11	[11]	76	F	GB	Abdominal Pain with Cholelithiasis	Regional LN; Liver	Cis + ETP → CBDCA + ETP; SST (no carcinoid sxs)	CCY	Died 5 mo
12	[19]	72	M	CBD	Jaundice	Liver	Cis + ETP	RHepLob+ extra-hepatic BD and PV resection after Percu/ Trans-hepatic PV Embo	Alive 7mo post-surgery

^a^Duration from diagnosis

*Ref*. Reference number

*M* male, *F* female

*GB* gallbladder, *CBD* common bile duct

*hx* history, *CCY* Cholecystectomy, *LN* lymph node, *LR* Liver resection

*NS* not specified, *HJ* hepaticojejunostomy

*Cis* Cisplatin, *ETP* Etoposide, *CAV* Cyclophosphamide/Adriamycin/Vincristine, *RT* radiation therapy, *TXT* Docetaxel, *CBDCA* Carboplatin, *5-FU* 5-fluorouracil, *EPR* epirubicin, *SST* Somatostatin analog

*Unresect* unresectable, *LND* lymphadenectomy, *RHepTriseg* right hepatic trisegmentectomy, *cerebell +γ-knife* cerebellectomy with γ-knife irradiation, *RHepLob* Right hepatic lobectomy, *BD* Bile Duct, *PV* Portal Vein, *Percu/Trans-hepatic* Percutaneous Transhepatic, *Embo* embolization

*mo* month, *Bx* biopsy, *sxs* symptoms

## Results

Based on our exhaustive search, we only found 12 cases of hepatobiliary pure large cell neuroendocrine carcinoma to date with 8 reported in the gallbladder, 3 in the common bile duct, and one reported in both gallbladder and biliary tract (Table 1). Papotti et al. [6] reported the first case of primary LCNEC of the gallbladder in 2000 while Sastomi et al. [8] reported the first primary common bile duct LCNEC in 2013. The median age at presentation was 66 with a range of 55 to 76 with an equal male-to-female ratio of 1:1. For primary gallbladder LCNEC, the median age at presentation was 64 (range 55–76) while for primary biliary duct LCNEC the median age was 73 (range 67–76). All patients either presented with metastases or quickly developed them with the most common site being regional lymph nodes and the liver. The most common symptoms at presentation were abdominal pain (58%), jaundice (41%), nausea (8%), and abdominal fullness (8%). One patient presented with exophthalmos secondary to metastatic disease.

Seven of the 12 patients underwent surgery, most commonly with cholecystectomy, bile duct excision, and/or liver resection. Eleven of the 12 patients received chemotherapy. Three received an unspecified regimen, 5 received cisplatin (or carboplatin) with etoposide, 1 received cisplatin (and later carboplatin) with docetaxel, and one received 5-fluorouracil, epirubicin, and cisplatin. In case 9, the patient was unable to undergo chemotherapy due to rapid progression of disease and died shortly after undergoing surgery. Unfortunately the prognosis of hepatobiliary LCNEC appears poor with an 83% (10 out of 12) mortality within 5 years. The median survival was 11 months after initial diagnosis with a range of 21 days to 69 months. The published reports identified two surviving patients who were treated with surgery and adjuvant cisplatin + etoposide and were, at the time, 7 months post-operative (case 4 and 12). Shimono et al. [17] reported a case with the longest survival in which a patient lived for 69 months using a multi-modal treatment therapy with multiple, and differing, chemotherapy regimens, surgical intervention, and radiation therapy.

## Discussion

Primary neuroendocrine carcinomas of the gallbladder and the biliary tract are rare with pure large cell neuroendocrine carcinomas being exceedingly rare and having a particularly poor prognosis [9, 10]. Papotti et al. [6] described the first case occurring in 2000 and since then, there have only been 11 more cases identified in literature. The rare nature of this disease may be due, in part, to the fact that neuroendocrine cells are not normally a part of the gallbladder and biliary tract mucosa. It has been postulated that these cancers may originate in the setting of an undifferentiated stem cell or in the setting of chronic inflammation leading to metaplasia and subsequent malignant conversion [11–13]. Large cell neuroendocrine carcinomas are a high grade heterogeneous group of tumors that can be distinguished by their histology and immunohistochemical staining. These cancers have positive immunohistochemical neuroendocrine markers which include synaptophysin, chromogranin A, CD56, and/or NSE.

LCNEC of the gallbladder and biliary tract present with symptoms similar to common adenocarcinoma, with non-specific symptoms of abdominal pain, jaundice, and generalized abdominal discomfort, making it difficult to diagnose pre-operatively [10, 13, 18]. Radiographically LCNEC appears similar to other neoplasms originating in the same region and blood tests do not differentiation status [11, 13]. Diagnosis is usually made after cholecystectomy for symptomatic cholelithiasis with histo-pathological and immunohistochemical analysis as outlined above. Pre-operative brush biopsies often provide high false-negative results and therefore are of little use [12]. These tumors are highly aggressive and present with, or quickly develop metastasis, most commonly to regional lymph nodes and the liver, which results in an overall poor prognosis [10, 13–15]. Prognosis appears to be similar to or worse than that observed with SCNEC since it is not as responsive to chemotherapy as SCNECs and most patients present with advanced disease [10, 15]. The median survival after initial diagnosis was only 11 months with a range of 21 days to 69 months. Because of the rarity of the disease, optimal management is still unclear and despite current therapies that have been utilized, the survival time continues to be short aside from one reported case by Shimono et al. [17].

Distiniguishing LCNEC of the gallbladder and biliary tract from adenocarcinoma is particularly important because the treatment modality, chemotherapy agents, and options for supplemental treatments differ [6, 17]. As with gallbladder adenocarcinoma, complete surgical resection offers the best chance for a cure and seems to prolong life although recurrence rates continue to be high [13, 15, 20]. Seven of the 12 patients underwent surgery, most commonly with cholecystectomy, bile duct excision, and/or liver resection. Importantly, in contrast to the treatment plan for other cancers, surgery should be considered in patients with stage I-III LCNEC [21]. In the 5 cases that did not undergo surgery due to the unresectable nature of their disease with multiple metastases, all did receive chemotherapy. Systemic chemotherapy is a mainstay of treatment along with surgery, as surgery alone does not appear to be sufficient [11, 21]. In addition, those that did not receive chemotherapy had a worse outcome as noted in case 9 by Sasatomi et al. [8] who died after 21 days from diagnosis. Although LCNEC is technically considered as non-small cell carcinoma, the treatment regimen is often similar to small cell lung cancer due to its aggressive nature as noted in Sun et al. [21, 22]. As with LCNEC of the lung, first line adjuvant chemotherapy with cisplatin or carboplatin and etoposide provide relatively good responses [11, 17, 19, 21, 23], and were only slightly worse than the results observed in SCNEC with similar regimens [21]. Five of the reported cases reported use of this regimen, and two of these remained alive 16 and 7 months after surgery respectively (case 4 & 12) with a median overall survival of 5 months. Other regimens included cisplatin (and later carboplatin) with docetaxel, and one patient received 5-fluorouracil, epirubicin, and cisplatin; both treatment regimens provided relatively good responses with survivals of 22 months and 12 months respectively.

Additional treatment modalities have yet to be thoroughly evaluated but may show some promise. For example, Shimono et al. [17] reported the longest surviving patient who lived 69 months after initial diagnosis with a multimodal treatment plan. This included consecutive neoadjuvant radiation and chemotherapy via hepatic artery infusion with cisplatin and etoposide followed by a combination regimen with cyclophosphamide/ adriamycin/vincristine, radical surgical resection after tumor shrinkage, adjuvant systemic chemotherapy with cisplatin and etoposide to prevent and manage metastases, and gamma-knife irradiation for brain metastasis [17]. Although most NETs are unresponsive to radiation therapy, this case indicates that radiation may prove to be a useful tool in management of locally advanced biliary LCNEC [17]. Buscemi et al. [11] also used a somatostatin analog in the setting of a patient without carcinoid syndrome with unclear benefit. Future studies are looking into molecular biomarkers which might open avenues for targeted and immunotherapies. With increasing access to techniques like next generation sequencing we now have some insights into the mutational profile of LCNEC. Much of this data however stems from thoracic and gastrointestinal LCNEC. Miyoshi et al. performed whole exome gene sequencing on 78 LCNEC samples. They compared the genomic alteration to 141 SCLC samples. Authors found inactivation TP53 mutation in 71% of samples and RB1 mutation in 26% samples. Mutations in PI3K/AKT/mTOR pathway was noted in 15% samples. Other molecular alteration of significance reported were 1) KRAS (6%), 2) FGFR1 (5%), 3) KIT (4%) and 4) ERBB2 (4%) [24]. Rekhtman et al. reported their findings of next generation sequencing of 45 pulmonary LCNEC cases. Mutation in *TP53* was reported in 78% samples and RB1 was mutated in 38% samples. Other molecular aberrations of significance were *STK11* in 33%, *KEAP1* in 31%, and *KRAS* in 22% [25].

Similar findings are noted in poorly differentiated pancreatic neuroendocrine carcinoma. Yachida et al. reported high prevalence of TP 53 (95%) and RB 1 (74%) mutation in poorly differentiated NECs of pancreas (LCNEC and small cell carcinoma). TP 53 was found to be a prognostic marker of poor outcome [26]. In comparison low grade NETs or carcinoid tumors follow an indolent course [27]. It is not surprising that low grade Nets have relatively fewer mutations and have virtually no mutation in TP 53 and RB 1 [26]. This is an evolving arena and might impact future classification of neuroendocrine neoplasms. Expanding knowledge about driver mutations can potentially translate into molecularly targeted therapeutic options.

Lastly, there has been some early data regarding role of immunotherapy in high grade neuroendocrine carcinomas like LCNEC. Dr. Patel presented data from a small phase II study looking into anti-tumor activity of ipilimumab and nivolumab in rare tumors. The high-grade neuroendocrine carcinoma cohort had an overall response rate of 44%. These findings are very promising and needs to be validated in larger phase III trials [28].

## Conclusions

Overall, complete surgical excision with systemic chemotherapy appears to be the mainstay treatment. If the cancer is unresectable due to multiple metastases, medical management with systemic chemotherapy is the primary modality [11]. Unfortunately, the prognosis of hepatobiliary LCNEC appears poor with 83% (10 out of 12) mortality within 5 years, and a median survival of 11 months after initial diagnosis with a range of 21 days to 69 months despite treatment modalities used. Due to the scarcity of this diagnosis, more studies will need to be done to determine the optimal treatment for LCNEC, however, we believe a multimodal regimen, molecular sequencing and immunotherapy holds the most promise for the future.

## Data Availability

All data generated or analyzed during this study are included in this published article (Table 1).

## Abbreviations

HPF: High power field
LCNED: Large cell neuroendocrine carcinomas
MiNEN: Mixed neuroendocrine-non-neuroendocrine neoplasms
NEC: Neuroendocrine carcinomas
NEN: Neuroendocrine neoplasms
NET: Neuroendocrine tumors
NSE: Neuron-specific enolase
SCNEC: Small cell neuroendocrine carcinomas
WHO: World Health Organization
